# Global research trends and emerging frontiers of gut microbiota–drug interactions: a bibliometric and visualization analysis (2003–2025)

**DOI:** 10.3389/fmicb.2026.1957920

**Published:** 2026-09-25

**Authors:** Yingxian Dong, Longxi He, Heng Du

**Affiliations:** 1 Department of Thoracic Surgery, West China Hospital, Sichuan University, Chengdu, Sichuan, China; 2 Lung Cancer Center, West China Hospital, Sichuan University, Chengdu, Sichuan, China; 3 Department of Pharmacy, West China Hospital, Sichuan University, Chengdu, China

**Keywords:** bibliometric analysis, drug metabolism, gut microbiota, pharmacomicrobiomics, precision medicine

## Abstract

**Background:**

The gut microbiota can alter drug metabolism, efficacy, and toxicity, while medications can reciprocally reshape microbial communities. However, the structure and maturation of research specifically addressing these bidirectional interactions have not been comprehensively mapped.

**Methods:**

The Web of Science Core Collection (WoSCC) was the sole source for bibliometric mapping because it provides standardized cited-reference metadata. PubMed was used only for complementary search-strategy validation. We analyzed 9,242 English-language articles and reviews published from 2003 through 2025 using VOSviewer, CiteSpace, and bibliometrix. A seeded random sample of 100 WoSCC records was assessed at title/abstract level to evaluate search specificity.

**Results:**

The 9,242-publication corpus showed sustained growth, reaching 2,160 publications in 2025. For descriptive comparison, the study period was divided *a priori* into foundational discovery (2003–2013), translational acceleration (2014–2020), and rapid expansion (2021–2025); these intervals were not statistically estimated change points. Keyword co-occurrence yielded five clusters: pharmacomicrobiomics/drug metabolism, microbiota–immune oncology, metabolic disease, gastrointestinal/inflammatory disorders, and the gut–brain axis. Thematic and burst analyses additionally highlighted natural products, network pharmacology, immunotherapy, and microbiota-directed interventions as recent or rapidly developing topics. In the 100-record specificity audit, 82 records were directly relevant at title/abstract level.

**Conclusion:**

This large-scale bibliometric analysis maps the development of gut microbiota–drug interaction research while distinguishing established clusters from topics identified as recent frontiers. The findings support growing translational interest but should not be interpreted as evidence of clinical efficacy or research quality.

## 1 Introduction

The gut microbiota comprises the microorganisms inhabiting the gastrointestinal tract, whereas the gut microbiome refers more broadly to these organisms, their genes, and their functional products. In this analysis, the controlled concept was gut microbiota; microbiome and intestinal microbiota were retained as search synonyms to capture indexing variation. Gut microorganisms can directly transform xenobiotics through reduction, hydrolysis, deconjugation, and other enzymatic reactions, thereby altering drug exposure, efficacy, or toxicity (Kang et al., 2013; Li et al., 2016; Swanson, 2015; Zimmermann et al., 2019; Heirali et al., 2023; Dhurjad et al., 2022). Conversely, antibiotics and many non-antibiotic medications can alter microbial community structure and function (Weersma et al., 2020; Wan and Zuo, 2022; Maier et al., 2018; Nagata et al., 2022).

Pharmacomicrobiomics is the study of how inter-individual variation in the microbiome influences drug disposition, response, and toxicity, together with the reciprocal effects of drugs on microbial ecology (Dikeocha et al., 2022; Spencer et al., 2021; Guthrie and Kelly, 2019; Nagata et al., 2022; Colotti and Rinaldi, 2020). This bidirectional framework connects microbial drug metabolism, medication-induced dysbiosis, biomarker discovery, and the development of microbiota-directed strategies intended to improve therapeutic outcomes.

Prior narrative reviews have synthesized individual mechanisms and disease settings (Kang et al., 2013; Li et al., 2016; Weersma et al., 2020; Wan and Zuo, 2022; Guthrie and Kelly, 2019), and bibliometric studies have mapped broader microbiome topics or disease-specific associations. Recent multi-database work has also illustrated the value of separating bibliometric mapping from clinically oriented synthesis (Zhou et al., 2026). A dedicated map of gut microbiota–drug interactions across pharmacological domains is nevertheless needed to clarify the field’s intellectual structure, analytical themes, and translational maturity without conflating citation prominence with clinical evidence.

We therefore conducted a large-scale bibliometric analysis of publications from 2003 through 2025. The start year was selected because it precedes the earliest sustained annual output in the retrieved corpus and captures the transition from sparse microbiota–drug reports to modern sequencing-enabled research. The end year was the most recent complete publication year at the July 20, 2026 search date; 2026 records were excluded to avoid incomplete-year bias. We aimed to characterize publication growth, collaboration and co-citation structures, keyword clusters, thematic evolution, and recent research frontiers. Our specific contribution is the integration of structural bibliometric mapping with a study-design-based appraisal of the ten most co-cited references and an explicit distinction between established clusters and emerging topics.

## 2 Methods

### 2.1 Search strategy and selection criteria

We searched WoSCC and PubMed on July 20, 2026 for publications dated January 1, 2003 through December 31, 2025. The complete database-specific queries are provided in Supplementary Table 1. WoSCC was the sole analytical database because its cited-reference and citation metadata support reproducible co-citation and burst analyses. PubMed returned 14,792 hits and was used only to validate the breadth of the search concepts and identify potentially missed terminology; PubMed records were not merged into the bibliometric dataset. The WoSCC search returned 9,428 records, of which 186 records carried a 2026 publication year and were excluded, leaving 9,242 English-language articles and reviews for analysis. We excluded meeting abstracts, editorials, corrections, and other document types. A seeded simple random sample of 100 included records (seed 20260831) was reviewed at title/abstract level against a prespecified criterion requiring an explicit gut-microbiota concept, a drug or therapeutic exposure, and an interaction-related outcome (metabolism, efficacy, toxicity, response, or microbiota modulation). Ninety-two records met this direct-relevance criterion; the remaining 8 illustrate the sensitivity–specificity trade-off of the broad search.

### 2.2 Bibliometric analysis

VOSviewer v1.6.20 was used for full-counting co-authorship, co-occurrence, and co-citation networks (van Eck and Waltman, 2010). Association-strength normalization and the default VOS clustering algorithm were used; network-specific minimum thresholds and attraction/repulsion settings are listed in the Supplementary methods. CiteSpace v6.3.R1 was used with one-year time slices (2003–2025), g-index node selection (*k* = 25), pathfinder pruning, and burst detection for cited references and keywords (Chen, 2006). Bibliometrix v4.1 was used for descriptive indicators, thematic mapping, and thematic evolution (Aria and Cuccurullo, 2017). The thematic-evolution intervals (2003–2013, 2014–2020, and 2021–2025) were defined *a priori* for descriptive comparison and were not inferred as statistical change points. Author records were standardized using full names when available, initials, affiliations, and persistent identifiers; ambiguous common-name records were not forcibly merged. Institution and keyword variants were harmonized with project thesauri, while original exports and thesaurus files were retained for auditability.

### 2.3 Evidence level stratification

The ten most co-cited references were classified descriptively by study design (*in vitro*, animal, human observational, interventional clinical, methodological/metagenomic, or review). The classification was used to characterize the types of work forming the field’s intellectual base, not to grade therapeutic efficacy. The classification was performed independently by two authors with a reconciliation step. Because co-citation prominence is not a representative sample of the entire evidence base, no population-level inference about clinical evidence was made.

## 3 Results

### 3.1 Publication trends

The final WoSCC dataset comprised 9,242 publications from 2003 through 2025 (Figure 1A). Annual output increased from isolated publications in the early years to 2,160 publications in 2025. For descriptive interpretation, we used three prespecified intervals: foundational discovery (2003–2013), translational acceleration (2014–2020), and rapid expansion (2021–2025). These labels summarize changes in publication volume and thematic emphasis but do not represent statistically estimated breakpoints. The publication curve therefore is presented descriptively rather than as evidence that particular scientific events caused growth.

**Figure 1:**
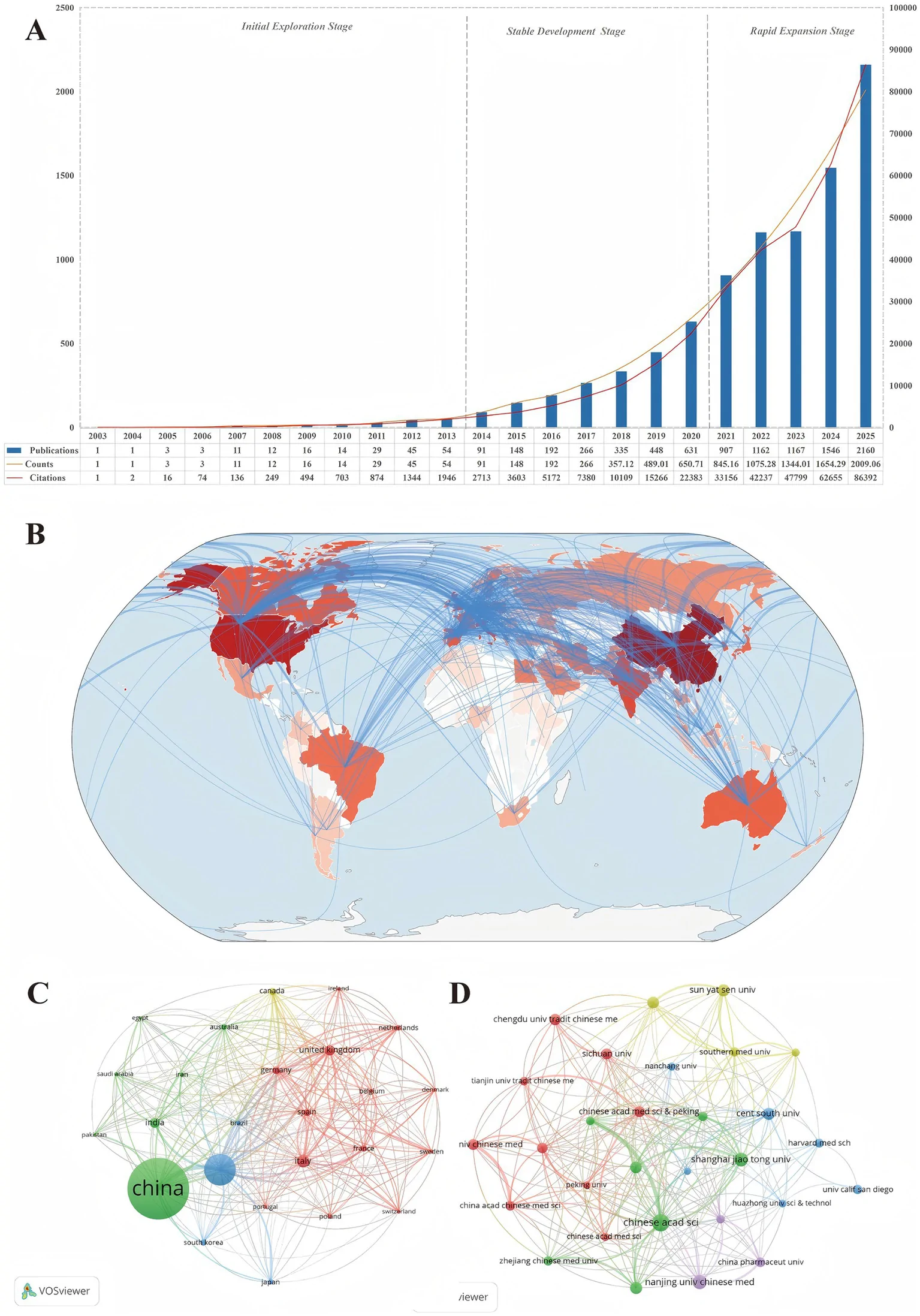
Publication trends and collaboration structure in gut microbiota–drug interaction research. **(A)** Annual WoSCC output for the 9,242 publications dated 2003–2025. Shaded bands denote the prespecified descriptive intervals 2003–2013, 2014–2020, and 2021–2025; they are not statistically estimated breakpoints. **(B)** Geographic publication distribution. **(C)** Country co-authorship network (full counting; minimum 80 documents; association-strength normalization; attraction/repulsion 2/−1). **(D)** Institution co-authorship network (full counting; minimum 60 publications; attraction/repulsion 3/−2). Node size represents output, line width represents link strength, and color denotes VOSviewer clusters.

### 3.2 Countries and institutions

The field was geographically broad (Figures 1B,C). China contributed the largest number of publications in the filtered dataset, followed by the United States; the collaboration network placed both countries among the most connected nodes. Citation totals and citations per publication are reported as descriptive measures only. They are affected by publication year, document type, subject mix, and citation window and should not be interpreted as direct measures of research quality.

Chinese institutions remained prominent in the publication network (Figure 1D; Supplementary Table 2), alongside major institutions in the United States and Europe. This distribution may partly reflect differences in research capacity, indexing coverage, national research priorities, and the growth of traditional-medicine-related microbiome research; the bibliometric data do not establish a causal explanation.

### 3.3 Journals and co-cited journals

The literature was distributed across pharmacology, microbiology, nutrition, and gastroenterology journals (Figure 2A). Frontiers in Pharmacology, International Journal of Molecular Sciences, Frontiers in Microbiology, Nutrients, and Phytomedicine were among the most productive sources. Impact factors and quartiles correspond to the 2024 Journal Citation Reports edition and are descriptive journal-level attributes rather than article-quality measures.

**Figure 2:**
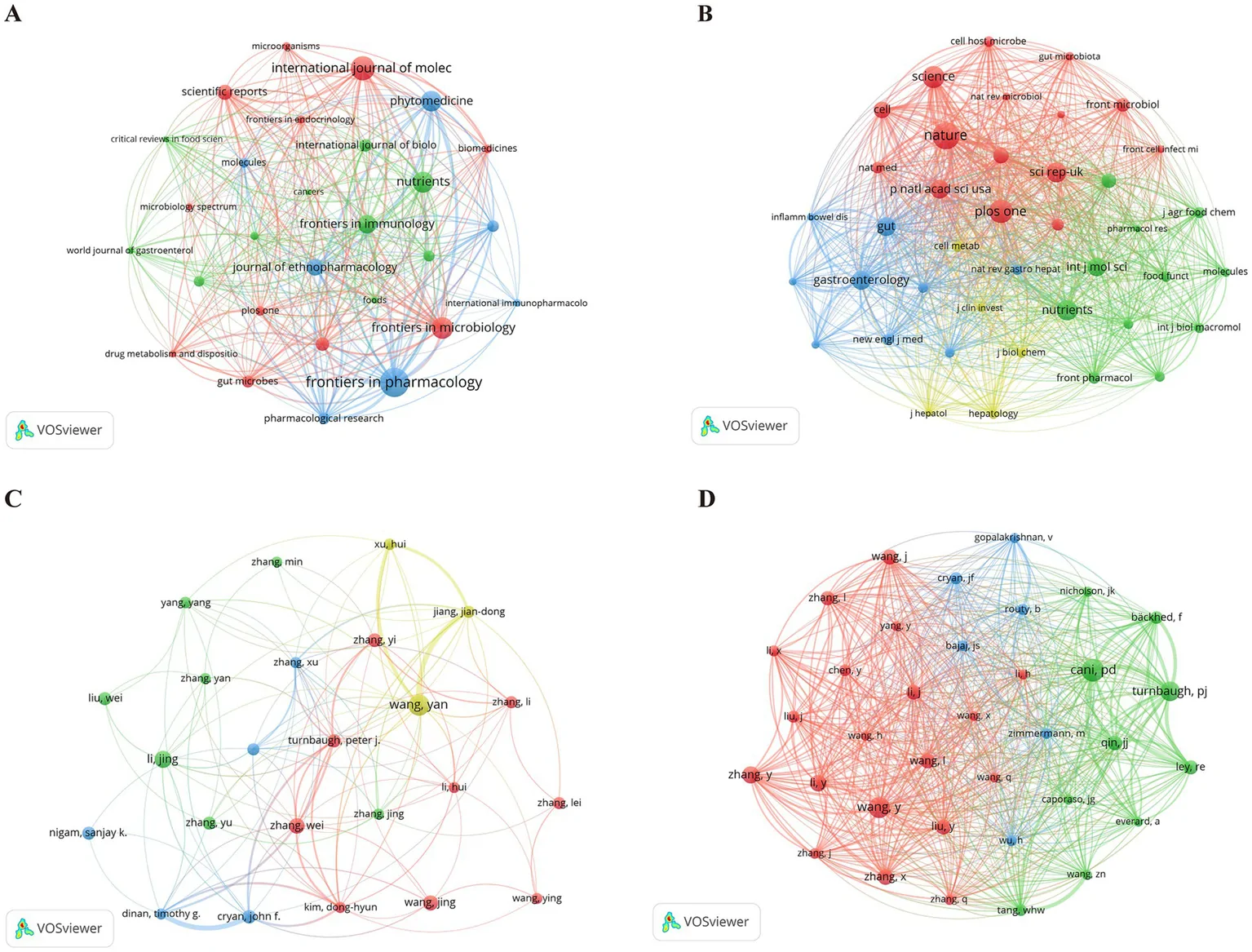
Journal and author networks. **(A)** Source publication network (full counting; minimum 40 documents; attraction/repulsion 3/−4). **(B)** Cited-source co-citation network (minimum 3,000 co-citations; attraction/repulsion 2/−1). **(C)** Author co-authorship network (minimum 20 documents; attraction/repulsion 4/−3). **(D)** Cited-author co-citation network (minimum 400 co-citations; attraction/repulsion 4/−3). Nodes represent sources or authors, line width represents link strength, and colors indicate algorithmically derived clusters. Author-name homonymy may affect surname-and-initial records.

Co-citation analysis (Figure 2B, Supplementary Table 3) reveals the field’s intellectual backbone. Nature is the most co-cited journal (15,964 co-citations; IF = 50.5, Q1), followed by PLOS ONE (13,300; IF = 2.9, Q2), Science (12,220; IF = 44.7, Q1), and Nutrients (11,046; IF = 5.9, Q1). Specialty journals like Gut (10,201; IF = 24.5, Q1), and Gastroenterology (10,184; IF = 25.7, Q1) form the core disciplinary foundation. The dense co-citation network suggests a well-integrated knowledge base spanning microbiology, gastroenterology, immunology, and drug development.

### 3.4 Authors and co-cited authors

Author productivity was evaluated after conservative name cleaning (Figure 2C). Because initials and common surnames can merge distinct individuals, rankings involving names such as Wang, Zhang, and Li should be interpreted cautiously. Persistent identifiers and affiliations were used when available, but ambiguous records were not forcibly consolidated.

The co-cited-author network (Figure 2D; Supplementary Table 4) identifies influential intellectual lineages rather than verified individual identities. Patrice D. Cani and Wang. Y were prominent nodes. Entries represented only by surname and initials, especially common East Asian names, may contain homonymy artifacts; this limitation applies to both the Results and Discussion.

### 3.5 Top co-cited references and evidence levels

The ten most co-cited references (Figures 3A,B; Supplementary Table 5) included foundational metagenomic, mechanistic, animal, and human observational studies. The leading references were Turnbaugh et al. (2006), Routy et al. (2018), Qin et al. (2010), David et al. (2014), Gopalakrishnan et al. (2018), Maier et al. (2018), Qin et al. (2012), Sivan et al. (2015), Zimmermann et al. (2019), and Vétizou et al. (2015). Figure 3A is a reference co-citation network; Figure 3B displays statistically detected periods of unusually rapid citation increase for cited references.

**Figure 3:**
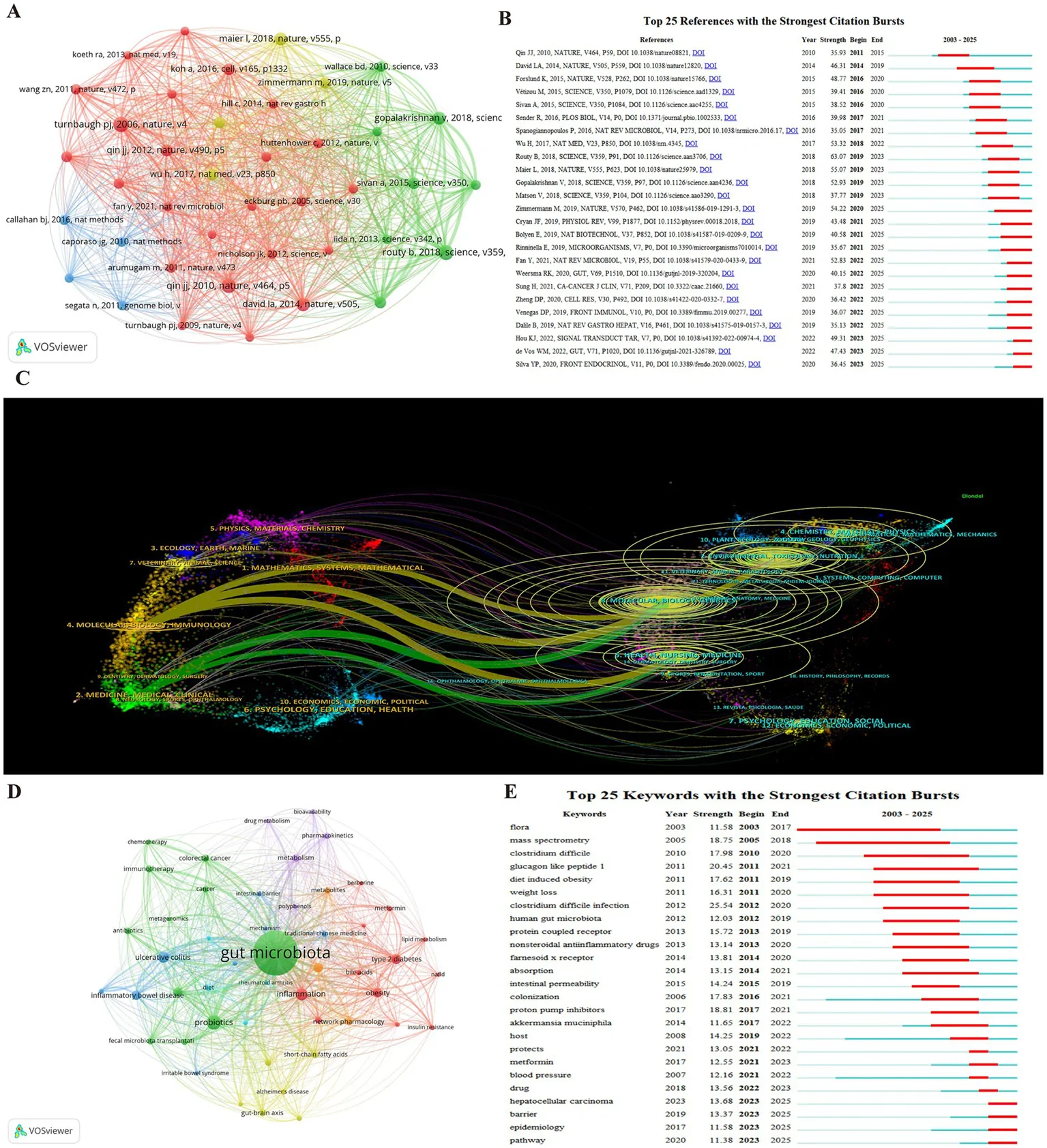
Reference co-citation and keyword analyses. **(A)** Reference co-citation network (minimum 200 co-citations; attraction/repulsion 2/−4). **(B)** Top 25 cited references with the strongest citation bursts detected in CiteSpace using one-year slices, g-index *k* = 25, and pathfinder pruning. Red segments denote statistically detected periods of unusually rapid citation increase; blue segments denote baseline periods. **(C)** Dual-map overlay of citing and cited journals. **(D)** Keyword co-occurrence network based on author and index keywords (minimum 65 occurrences; full counting; association-strength normalization; attraction/repulsion 2/0). **(E)** Top 25 keyword bursts; Red segments denote periods of unusually rapid increase in keyword usage, not citation counts.

Study-design classification showed that this highly co-cited set spans metagenomic or methodological studies, animal and *in vitro* experiments, human observational cohorts, and translational clinical investigations. This mixture indicates that the intellectual foundation is predominantly mechanistic and observational, while also containing clinically oriented work. Because the set comprises only ten highly co-cited references, it cannot estimate the proportion or quality of clinical evidence in the wider contemporary literature. The dual-map overlay summarizes links between citing and cited journal disciplines (Figure 3C).

### 3.6 Keyword clustering

Keyword co-occurrence analysis (Figure 3D; Supplementary Table 6) yielded five clusters: (1) pharmacomicrobiomics and drug metabolism; (2) the microbiota–immune-oncology axis; (3) metabolic disease; (4) gastrointestinal and inflammatory disorders; and (5) the gut–brain axis. Natural-product terms did not constitute a separate fifth co-occurrence cluster. Instead, natural products, traditional Chinese medicine, and network pharmacology became prominent in the thematic and temporal analyses and are therefore discussed as a cross-cutting recent direction. The top 25 keyword bursts and their temporal intervals are shown in Figure 3E.

### 3.7 Thematic evolution

Figure 4A traces themes across the same prespecified intervals used throughout the manuscript: 2003–2013, 2014–2020, and 2021–2025. The early interval included microbiota, probiotic, metabolic, and immune-related terms; the middle interval included gut microbiota, probiotics, inflammation, inflammatory bowel disease, the gut-brain axis, and irritable bowel syndrome; and the most recent interval included probiotics, gut microbiota, inflammation, the gut-brain axis, and immunotherapy. The alluvial links indicate continuity and reorganization of terms, not causal progression.

**Figure 4:**
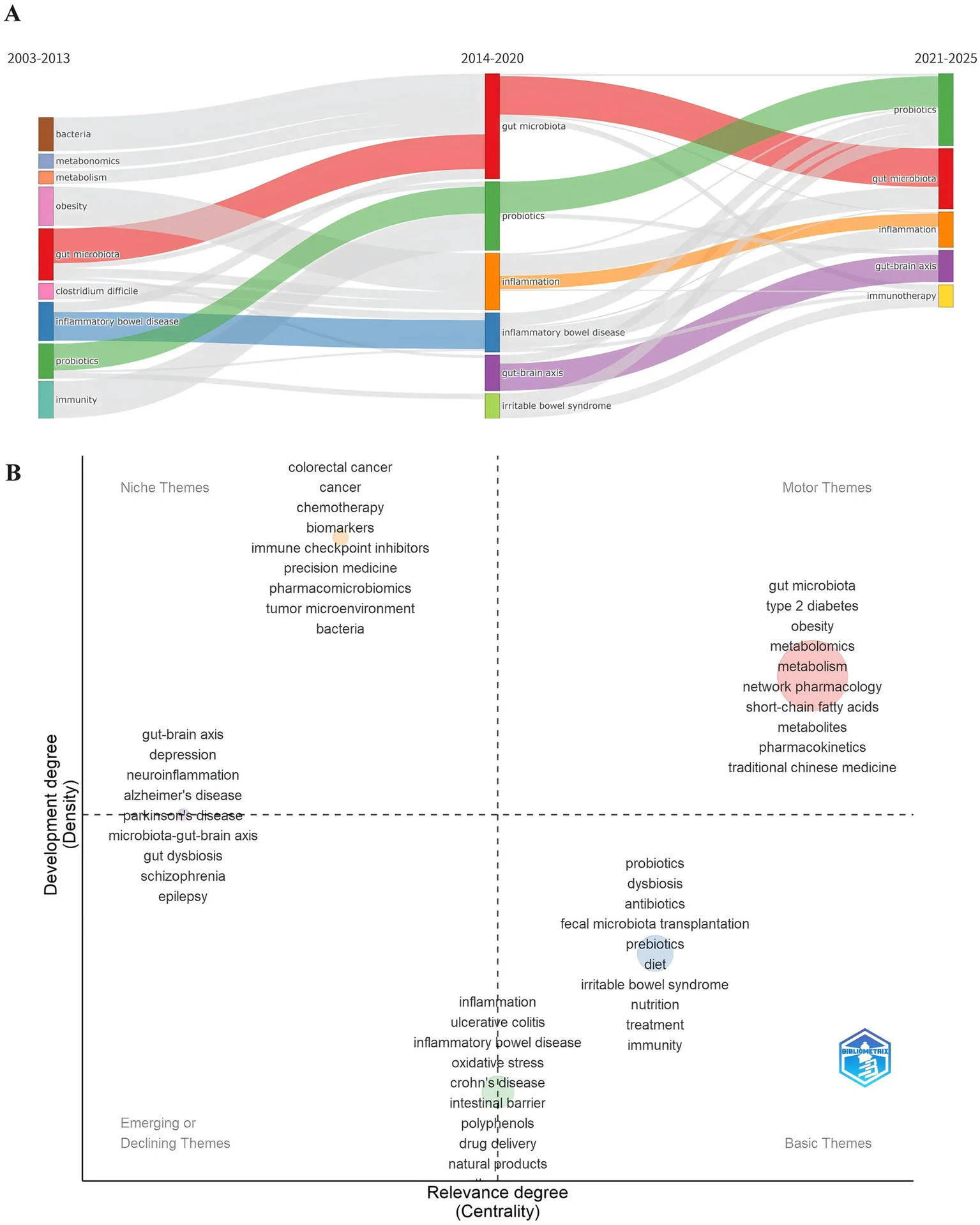
Thematic analysis in bibliometrix: **(A)** thematic evolution across the prespecified intervals 2003–2013, 2014–2020, and 2021–2025. Links show continuity of shared terms between intervals. **(B)** Thematic map based on keyword co-occurrence. Centrality (horizontal axis) represents relevance to the broader field and density (vertical axis) represents internal development. The upper-right, upper-left, lower-left, and lower-right quadrants denote motor, niche, emerging/declining, and basic themes, respectively. Quadrant position alone cannot distinguish an emerging theme from a declining one.

The thematic map (Figure 4B) places the large gut-microbiota/metabolic theme, including network pharmacology, in the motor-theme quadrant. Probiotics, dysbiosis, antibiotics, fecal microbiota transplantation, diet, and related terms occupy the basic-theme quadrant. The inflammatory bowel disease/inflammation theme lies near or within the emerging/declining region, whereas immunotherapy and pharmacomicrobiomics form more specialized, developed themes. Quadrant membership describes centrality and density at the analysis time point; it does not by itself distinguish emergence from decline.

## 4 Discussion

Together, the 9,242 publications describe a field that has expanded from sparse mechanistic observations toward broader pharmacological and translational questions. Bibliometric associations identify changes in emphasis but do not demonstrate that specific discoveries caused publication growth or that highly cited topics are clinically validated.

### 4.1 Historical arc and global patterns

Publication output rose across the three prespecified descriptive intervals. Growth coincided with advances in sequencing, mechanistic studies of microbial drug metabolism, and increasing interest in microbiota–immune interactions (Zimmermann et al., 2019; Weersma et al., 2020; Guthrie and Kelly, 2019; Nagata et al., 2022), but causal attribution is not possible from publication counts. China led publication volume, while the United States occupied a central position in collaboration and citation networks. Differences in citation averages are not direct quality comparisons because citation age, document type, field, and citation window were not normalized.

### 4.2 Intellectual foundations

Reference and journal co-citation analyses show interdisciplinary links among microbiome science, pharmacology, gastroenterology, immunology, and metabolism. Prominent authors and references should be interpreted as influential within this corpus rather than as exhaustive measures of individual contribution. In particular, surname-and-initial records may conflate different authors.

### 4.3 Where the field is heading

The three areas illustrated in Figure 5—bacterial drug metabolism, microbiota–immune checkpoint modulation, and natural product–microbiota interactions—were selected as a narrative synthesis rather than as the three largest keyword clusters. Selection required convergence of at least two signals: prominence or recent appearance in thematic evolution, presence in keyword or reference bursts, and mechanistic or translational relevance documented by highly co-cited or recent studies. Metabolic disease, gastrointestinal inflammation, and the gut–brain axis remain established clusters but were not chosen for the schematic because Figure 5 focuses on bidirectional drug–microbiota mechanisms with near-term translational implications.

**Figure 5:**
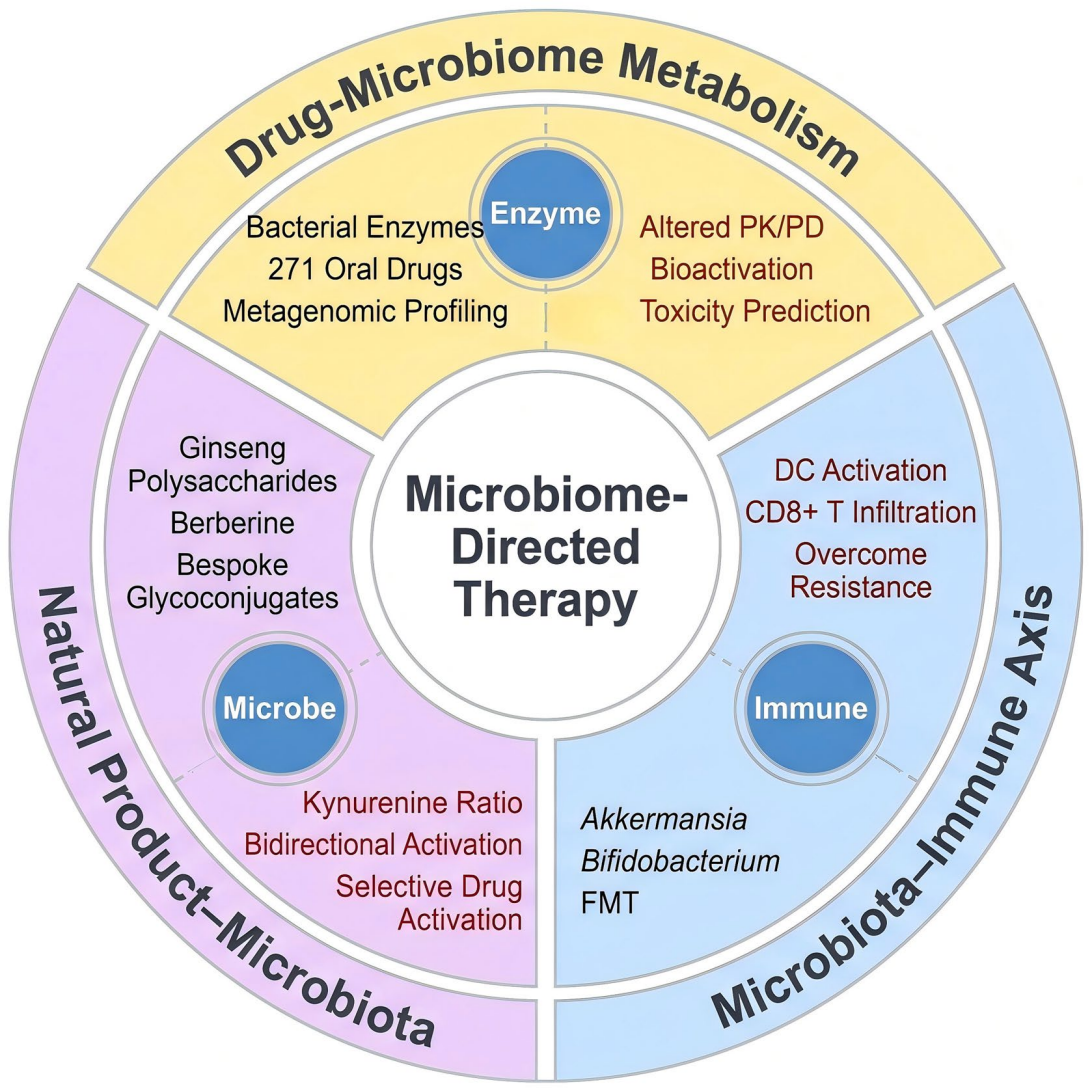
Narrative synthesis of three translationally relevant research directions. The schematic integrates (1) bacterial drug metabolism, (2) microbiota–immune checkpoint modulation, and (3) natural product–microbiota interactions. These directions were selected from convergent thematic-evolution, burst, and mechanistic-literature signals; This figure is not a direct bibliometric network output and does not imply that these are the three largest keyword clusters.

#### 4.3.1 Pharmacomicrobiomics and bacterial drug metabolism

At the most fundamental level, researchers are trying to figure out exactly how gut bacteria chemically modify drugs. Zimmermann et al. (2019) showed that roughly two-thirds of 271 tested oral drugs were metabolized by at least one gut bacterial strain in vitro. This finding demonstrates widespread bacterial drug-metabolizing capacity and its potential relevance to drug disposition. Subsequent pharmacokinetic modeling has begun to quantify the relative contributions of host and microbial metabolism (Zimmermann-Kogadeeva et al., 2020). The bacterial enzymes involved—reductases, hydrolases, acetyltransferases—could become therapeutic targets (Pant et al., 2023; Tan et al., 2024). Population-level metagenomic studies have characterized medication-associated changes in the gut microbiome, while experimental models provide tools for studying microbial drug metabolism (Nagata et al., 2022; Tan et al., 2024). This mechanistic work lays the groundwork for personalized pharmacomicrobiomic interventions (Guthrie and Kelly, 2019; Colotti and Rinaldi, 2020; Zhao et al., 2023).

#### 4.3.2 Microbiota-immune checkpoint modulation in cancer therapy

The intersection of gut-microbiome research and cancer immunotherapy is a clinically active direction. Routy et al. and Gopalakrishnan et al. associated gut-microbiome composition with response to PD-1/PD-L1 blockade in epithelial tumors and melanoma (Gopalakrishnan et al., 2018; Routy et al., 2018). *Akkermansia muciniphila* and *Bifidobacterium* spp. have been linked to antitumor immune activity in human cohorts and experimental models (Gopalakrishnan et al., 2018; Routy et al., 2018; Sivan et al., 2015). Early clinical studies further suggest that fecal microbiota transplantation from responding donors may restore sensitivity in a subset of immunotherapy-refractory melanoma patients (Baruch et al., 2021; Davar et al., 2021). These findings are promising but remain dependent on small cohorts, heterogeneous interventions, and prospective validation.

#### 4.3.3 Natural product-microbiota modulation and network pharmacology

A distinctive frontier lies at the intersection of traditional medicine, natural product chemistry, and gut microbiota research. Natural products can interact with the gut microbiota through multiple mechanisms (Zhao et al., 2023; Huang et al., 2022). Huang et al., for example, showed that ginseng polysaccharides alter gut microbiota composition and the kynurenine/tryptophan ratio, thereby potentiating anti-PD-1/PD-L1 immunotherapy (Huang et al., 2022). Similar work has been done with berberine, curcumin, and polyphenols, which modulate drug bioavailability and efficacy through bacterial transformation (Pant et al., 2023; Yang et al., 2023). The interactions go both ways: gut bacteria can activate or detoxify natural compounds, while these compounds shape microbial community structure (Weersma et al., 2020; Zhao et al., 2023). Recent advances in designing plant glycoconjugates that are selectively activated by specific bacterial enzymes in the gut offer a promising direction for targeted drug delivery (Ma et al., 2025). This convergence of pharmacomicrobiomics and natural products could provide a blueprint for next-generation microbiota-directed therapeutics (Wan and Zuo, 2022; Maseda and Ricciotti, 2020; Bai et al., 2022).

### 4.4 Challenges

The study-design profile of the ten most co-cited references shows that the field’s intellectual base includes substantial mechanistic, metagenomic, animal, and observational work. This observation should not be generalized to the entire evidence base or interpreted through a therapeutic evidence hierarchy. Key translational challenges include inter-individual microbial variability, incomplete standardization of microbiota-directed interventions, causal attribution, and long-term safety (Heirali et al., 2023; Guthrie and Kelly, 2019; Sumiyoshi et al., 2023). Future work should connect validated microbial enzymes and metabolites to prospective clinical designs and harmonized multi-omics workflows.

### 4.5 Limitations

This study has several limitations. First, bibliometric mapping used WoSCC alone; PubMed served only as a complementary query check, and studies indexed exclusively in PubMed, Scopus, Embase, CNKI, or other regional databases were not represented in the networks. Scopus was not merged because citation-reference formats, coverage, and indexing rules differ across platforms, and combining them without a validated harmonization pipeline could duplicate records and distort network weights. Second, the broad, sensitivity-oriented query produced 92 directly relevant records in a seeded 100-record title/abstract audit, indicating that some included records address therapy-related microbiota modulation without a narrowly defined pharmacological interaction. Third, English-language and database-indexing restrictions may underrepresent regional literature. Fourth, name and affiliation normalization cannot fully resolve homonymy, especially for common surnames. Fifth, raw citation averages were not normalized for publication age, document type, or field. Finally, thematic quadrants and burst statistics indicate structural or temporal prominence but do not by themselves prove that a topic is emerging, clinically effective, or causally responsible for growth.

## 5 Conclusion

This bibliometric analysis of 9,242 WoSCC publications from 2003 through 2025 maps the structure and development of gut microbiota–drug interaction research. Across three prespecified descriptive intervals (2003–2013, 2014–2020, and 2021–2025), five established keyword clusters were identified: pharmacomicrobiomics/drug metabolism, microbiota–immune oncology, metabolic disease, gastrointestinal/inflammatory disorders, and the gut–brain axis. Thematic evolution and burst analyses additionally highlighted natural products, network pharmacology, immunotherapy, and microbiota-directed interventions as recent or rapidly developing topics. These bibliometric patterns define priorities for mechanistic and prospective clinical research but do not substitute for direct evaluation of therapeutic efficacy.

## Data Availability

The analyzed WoSCC metadata, the filtered 2003–2025 dataset, database queries, thesaurus files, and seeded 100-record relevance-audit table are available from the corresponding author subject to Clarivate's data-licensing terms. PubMed was used only for complementary query validation. Software parameters are provided in the Supplementary materials. The software packages used in this study are publicly available: VOSviewer v1.6.20 (https://www.vosviewer.com), CiteSpace v6.3.R1 (https://citespace.podia.com), and bibliometrix R package v4.1 (https://www.bibliometrix.org). Custom analytical scripts are available from the corresponding author upon reasonable request.
