# Supplementary material for: Global research trends and emerging frontiers of gut microbiota–drug interactions: a bibliometric and visualization analysis (2003–2025)
Source: Front Microbiol. 2026 Sep 25;17:1957920. doi: 10.3389/fmicb.2026.1957920 (PMC13649975; doi:10.3389/fmicb.2026.1957920)
Supplement: Supplementary file 1 [file Supplementary_file_1.docx]

# Supplementary Materials

Global research trends and emerging frontiers of gut microbiota-drug interactions: a bibliometric and visualization analysis (2003–2025)

## Supplementary Figure 1: PRISMA Flow Diagram

The PRISMA flowchart illustrates the systematic screening process for gut microbiota-drug interactions publications. Records identified from WoSCC (n=9,662) and PubMed (n=14,792) were combined, duplicates removed, and records screened by title/abstract. After full-text assessment, 9,242 publications were included in the final bibliometric analysis.

**Supplementary Table 1. Complete Search Strategies for Database Retrieval**

| **Database** | **Time Span** | **Search String** |
| --- | --- | --- |
| Web of Science Core Collection (WoSCC) | 2003-01-01 to 2025-12-31 | TS=(("gut microbiota" OR "gut microbiome" OR "intestinal microbiota" OR "fecal microbiota") AND (drug* OR pharmaco* OR medication) AND (metabolism OR efficacy OR toxicity OR interaction* OR response OR modulation)) |
| PubMed (validation only) | 2003-01-01 to 2025-12-31 | (("gut microbiota"[Title/Abstract] OR "gut microbiome"[Title/Abstract] OR "intestinal microbiota"[Title/Abstract] OR "fecal microbiota"[Title/Abstract]) AND (drug*[Title/Abstract] OR pharmaco*[Title/Abstract] OR medication[Title/Abstract]) AND (metabolism[Title/Abstract] OR efficacy[Title/Abstract] OR toxicity[Title/Abstract] OR interaction*[Title/Abstract] OR response[Title/Abstract] OR modulation[Title/Abstract])) |

**Notes:**

Search date: July 20, 2026. WoSCC was the sole data source for bibliometric analysis (9,428 retrieved; 186 records from 2026 excluded; final n=9,242). PubMed (n=14,792) was used only for search-strategy validation and supplementary checking and was not merged with WoSCC records.

**Supplementary Table 2: The top 10 countries and organizations**

| **Rank** | **Country** | **Counts** | **Citations** | **Average Citation/Publications** | **Organization** | **Counts** | **Citations** |
| --- | --- | --- | --- | --- | --- | --- | --- |
| 1 | China | 4340 | 130769 | 30.13 | Chinese Acad Sci | 179 | 9156 |
| 2 | United States | 1860 | 126716 | 68.13 | Nanjing Univ Chinese Med | 140 | 3900 |
| 3 | Italy | 562 | 31139 | 55.41 | Shanghai Jiao Tong Univ | 136 | 9212 |
| 4 | United Kingdom | 469 | 45023 | 96.00 | Cent South Univ | 117 | 3522 |
| 5 | India | 445 | 11725 | 26.35 | Zhejiang Univ | 114 | 5070 |
| 6 | Germany | 315 | 26916 | 85.45 | Sun Yat Sen Univ | 113 | 4574 |
| 7 | Canada | 298 | 17822 | 59.81 | Shanghai Univ Tradit Chinese Med | 110 | 2864 |
| 8 | Spain | 291 | 17327 | 59.54 | Guangzhou Univ Chinese Med | 108 | 3452 |
| 9 | France | 261 | 23135 | 88.64 | Chengdu Univ Tradit Chinese Med | 107 | 3873 |
| 10 | South Korea | 246 | 12355 | 50.22 | Beijing Univ Chinese Med | 103 | 2510 |

**Supplementary Table 3: Top 10 journals and co-cited journals**

| **Rank** | **Journal** | **Counts** | **Citations** | **IFa** | **Qb** | **Co-cited journal** | **Co-citation** | **IFa** | **Qb** |
| --- | --- | --- | --- | --- | --- | --- | --- | --- | --- |
| 1 | Frontiers In Pharmacology | 277 | 7029 | 4.4 | Q1 | Nature | 15964 | 50.5 | Q1 |
| 2 | International Journal Of Molecular Sciences | 216 | 7929 | 4.9 | Q2 | Plos One | 13300 | 2.9 | Q2 |
| 3 | Frontiers In Microbiology | 188 | 4962 | 4.0 | Q2 | Science | 12220 | 44.7 | Q1 |
| 4 | Nutrients | 187 | 6671 | 5.9 | Q1 | Nutrients | 11046 | 5.9 | Q1 |
| 5 | Phytomedicine | 177 | 3775 | 7.9 | Q1 | Sci Rep-Uk | 10767 | 3.8 | Q2 |
| 6 | Frontiers In Immunology | 160 | 6881 | 5.7 | Q1 | Gut | 10201 | 24.5 | Q1 |
| 7 | Journal Of Ethnopharmacology | 137 | 3476 | 5.4 | Q1 | Gastroenterology | 10184 | 25.7 | Q1 |
| 8 | Scientific Reports | 121 | 5358 | 3.8 | Q2 | P Natl Acad Sci Usa | 9854 | 9.4 | Q1 |
| 9 | Frontiers In Cellular And Infection Microbiology | 104 | 3626 | 4.6 | Q1 | Int J Mol Sci | 9727 | 4.9 | Q2 |
| 10 | International Journal Of Biological Macromolecules | 97 | 2436 | 7.7 | Q1 | Cell | 8448 | 58.7 | Q1 |

**Supplementary Table 4: Top 10 authors and co-cited authors**

| **Rank** | **Author** | **Counts** | **Citations** | **Total Link Strength** | **Co-cited Authors** | **Co-citations** |
| --- | --- | --- | --- | --- | --- | --- |
| 1 | Wang, Yan | 47 | 1518 | 137 | Cani, PD | 1201 |
| 2 | Li, Jing | 37 | 946 | 18 | Wang, Y | 1071 |
| 3 | Zhang, Wei | 33 | 879 | 41 | Turnbaugh, PJ | 1002 |
| 4 | Wang, Jing | 31 | 709 | 13 | Zhang, Y | 793 |
| 5 | Zhang, Yi | 27 | 729 | 24 | Li, Y | 774 |
| 6 | Cryan, John F. | 27 | 6297 | 107 | Zhang, X | 724 |
| 7 | Liu, Wei | 26 | 933 | 5 | Qin, JJ | 707 |
| 8 | Nigam, Sanjay K. | 26 | 1764 | 3 | Liu, Y | 688 |
| 9 | Turnbaugh, Peter J. | 26 | 3067 | 77 | Wang, L | 679 |
| 10 | Zhang, Yu | 26 | 348 | 8 | Wang, J | 675 |

Note: Productive-author indicators were taken from the VOSviewer author co-authorship export. Because the supplied cited-author network text contained numeric edge identifiers without a name map, co-cited-author frequencies were reconstructed from cited first-author names in the CR field of the final 9,242-record WoSCC corpus.

**Supplementary Table 5: Top 10 co-cited references**

| **Rank** | **Co-cited reference** | **Citations** | **Title** | **Study design** | **Evidence context** | **DOI** |
| --- | --- | --- | --- | --- | --- | --- |
| 1 | turnbaugh pj, 2006, nature, v444, p1027, doi 10.1038/nature05414 | 414 | An obesity-associated gut microbiome with increased capacity for energy harvest | Animal metagenomic and mechanistic study | Preclinical | 10.1038/nature05414 |
| 2 | routy b, 2018, science, v359, p91, doi 10.1126/science.aan3706 | 404 | Gut microbiome influences efficacy of PD-1-based immunotherapy against epithelial tumors | Human cohorts with translational animal experiments | Translational (human and animal) | 10.1126/science.aan3706 |
| 3 | qin jj, 2010, nature, v464, p59, doi 10.1038/nature08821 | 394 | A human gut microbial gene catalogue established by metagenomic sequencing | Human metagenomic catalogue study | Human methodological | 10.1038/nature08821 |
| 4 | david la, 2014, nature, v505, p559, doi 10.1038/nature12820 | 344 | Diet rapidly and reproducibly alters the human gut microbiome | Controlled human dietary intervention | Human interventional | 10.1038/nature12820 |
| 5 | gopalakrishnan v, 2018, science, v359, p97, doi 10.1126/science.aan4236 | 337 | Gut microbiome modulates response to anti-PD-1 immunotherapy in melanoma patients | Prospective human observational cohort | Human observational | 10.1126/science.aan4236 |
| 6 | maier l, 2018, nature, v555, p623, doi 10.1038/nature25979 | 329 | Extensive impact of non-antibiotic drugs on human gut bacteria | In vitro high-throughput drug-bacteria screen | Preclinical in vitro | 10.1038/nature25979 |
| 7 | qin jj, 2012, nature, v490, p55, doi 10.1038/nature11450 | 311 | A metagenome-wide association study of gut microbiota in type 2 diabetes | Human metagenome-wide association study | Human observational | 10.1038/nature11450 |
| 8 | sivan a, 2015, science, v350, p1084, doi 10.1126/science.aac4255 | 301 | Commensal Bifidobacterium promotes antitumor immunity and facilitates anti-PD-L1 efficacy | Animal mechanistic study | Preclinical | 10.1126/science.aac4255 |
| 9 | zimmermann m, 2019, nature, v570, p462, doi 10.1038/s41586-019-1291-3 | 296 | Mapping human microbiome drug metabolism by gut bacteria and their genes | In vitro screening with animal pharmacokinetic validation | Translational preclinical | 10.1038/s41586-019-1291-3 |
| 10 | Vétizou M, 2015, Science, v350, p1079, doi 10.1126/science.aad1329 | 295 | Anticancer immunotherapy by CTLA-4 blockade relies on the gut microbiota | Animal mechanistic study with human-to-mouse validation | Translational (human and animal) | 10.1126/science.aad1329 |

Note: Co-citation counts were taken directly from the updated VOSviewer reference co-citation export. Study design and evidence context are descriptive classifications and are not clinical evidence-level ratings.

Supplementary Table 6: Keywords meeting the VOSviewer inclusion threshold (n=49)

| **Rank** | **Keywords** | **Counts** | **Rank** | **Keywords** | **Counts** |
| --- | --- | --- | --- | --- | --- |
| 1 | gut microbiota | 4321 | 26 | metformin | 117 |
| 2 | probiotics | 596 | 27 | neuroinflammation | 113 |
| 3 | inflammation | 428 | 28 | bile acids | 105 |
| 4 | ulcerative colitis | 377 | 29 | intestinal barrier | 98 |
| 5 | type 2 diabetes | 315 | 30 | alzheimer's disease | 98 |
| 6 | obesity | 301 | 31 | crohn's disease | 97 |
| 7 | inflammatory bowel disease | 292 | 32 | chemotherapy | 94 |
| 8 | metabolomics | 286 | 33 | insulin resistance | 93 |
| 9 | dysbiosis | 276 | 34 | lipid metabolism | 89 |
| 10 | metabolism | 201 | 35 | irritable bowel syndrome | 87 |
| 11 | gut-brain axis | 181 | 36 | polyphenols | 85 |
| 12 | immunotherapy | 173 | 37 | nutrition | 84 |
| 13 | network pharmacology | 172 | 38 | parkinson's disease | 83 |
| 14 | colorectal cancer | 169 | 39 | bioavailability | 83 |
| 15 | oxidative stress | 157 | 40 | drug metabolism | 83 |
| 16 | antibiotics | 152 | 41 | treatment | 82 |
| 17 | short-chain fatty acids | 151 | 42 | metabolic syndrome | 80 |
| 18 | fecal microbiota transplantation | 151 | 43 | immunity | 75 |
| 19 | pharmacokinetics | 149 | 44 | rheumatoid arthritis | 74 |
| 20 | cancer | 147 | 45 | metagenomics | 74 |
| 21 | metabolites | 146 | 46 | berberine | 73 |
| 22 | prebiotics | 140 | 47 | mechanism | 68 |
| 23 | depression | 139 | 48 | atherosclerosis | 67 |
| 24 | traditional chinese medicine | 124 | 49 | nafld | 66 |
| 25 | diet | 124 |  |  |  |

Note: The updated VOSviewer keyword export contained 49 terms meeting the minimum occurrence threshold (65); all are reported.

## Supplementary Methods: Software Parameters

**VOSviewer (v1.6.20):**

Country co-authorship: full counting, min 80 documents, attraction=2, repulsion=-1.

Organization co-authorship: full counting, min 60 publications, attraction=3, repulsion=-2.

Source co-authorship: full counting, min 40 documents, attraction=3, repulsion=-4.

Author co-authorship: full counting, min 20 documents, attraction=4, repulsion=-3.

Cited source co-citation: min 3000 citations, attraction=2, repulsion=-1.

Cited author co-citation: min 400 co-citations, attraction=4, repulsion=-3.

Reference co-citation: min 200 citations, attraction=2, repulsion=-4.

Keyword co-occurrence: author+index keywords, min 65 occurrences, attraction=2, repulsion=0.
