# Supplementary material for: Global research trends and emerging frontiers of gut microbiota–drug interactions: a bibliometric and visualization analysis (2003–2025)
Source: Front Microbiol. 2026 Sep 25;17:1957920. doi: 10.3389/fmicb.2026.1957920 (PMC13649975; doi:10.3389/fmicb.2026.1957920)
Supplement: Supplementary file 2 [file Supplementary_file_2.docx]

**Supplementary Methods**

*Global research trends and emerging frontiers of gut microbiota–drug interactions: a bibliometric and visualization analysis (2003–2025)*

# Supplementary Table 1. Complete search strategies

| Database | Period | Exact query |
| --- | --- | --- |
| WoSCC | 2003-01-01 to 2025-12-31 | TS=(("gut microbiota" OR "gut microbiome" OR "intestinal microbiota" OR "fecal microbiota") AND (drug* OR pharmaco* OR medication) AND (metabolism OR efficacy OR toxicity OR interaction* OR response OR modulation)) |
| PubMed (validation only) | 2003-01-01 to 2025-12-31 | (("gut microbiota"[Title/Abstract] OR "gut microbiome"[Title/Abstract] OR "intestinal microbiota"[Title/Abstract] OR "fecal microbiota"[Title/Abstract]) AND (drug*[Title/Abstract] OR pharmaco*[Title/Abstract] OR medication[Title/Abstract]) AND (metabolism[Title/Abstract] OR efficacy[Title/Abstract] OR toxicity[Title/Abstract] OR interaction*[Title/Abstract] OR response[Title/Abstract] OR modulation[Title/Abstract])) |

Search date: July 20, 2026. WoSCC was the sole analytical source. No cross-database merging or deduplication was performed.

# Supplementary Table 2. Search-specificity audit

| Classification | n | Operational criterion |
| --- | --- | --- |
| Directly relevant | 92 | Explicit gut-microbiota concept, drug/therapeutic exposure, and interaction outcome. |
| Not directly relevant | 8 | Did not clearly meet all three elements at title/abstract level. |
| Total | 100 | Simple random sample; seed 20260831. |

# Supplementary Table 3. Software parameters and data cleaning

| Component | Setting |
| --- | --- |
| VOSviewer | Full counting; association-strength normalization; country min 80 documents, organization min 60, source min 40, author min 20, cited source min 3,000, cited author min 400, cited reference min 200, keyword min 65 occurrences. |
| CiteSpace | One-year slices (2003–2025); g-index k=25; pathfinder pruning; separate burst detection for cited references and keywords. |
| bibliometrix | Author and index keywords after thesaurus harmonization; descriptive intervals 2003–2013, 2014–2020, 2021–2025; thematic-map interpretation based on centrality and density. |
| Name cleaning | Full names, initials, affiliations, and persistent identifiers used when available; ambiguous common-name records were not forcibly merged. |
| Institution/keyword cleaning | Institutional variants and keyword synonyms harmonized with retained project thesauri; original exports retained for auditability. |

# Supplementary Table 4. Study-design classification of the ten most co-cited references

| Reference | Study design |
| --- | --- |
| Turnbaugh et al., 2006 | Animal/metagenomic mechanistic study |
| Routy et al., 2018 | Human observational cohorts with translational animal experiments |
| Qin et al., 2010 | Human metagenomic catalogue/methodological study |
| David et al., 2014 | Controlled human dietary intervention |
| Gopalakrishnan et al., 2018 | Human observational clinical cohort |
| Maier et al., 2018 | In vitro high-throughput drug–bacteria screen |
| Qin et al., 2012 | Human observational metagenome-wide association study |
| Sivan et al., 2015 | Animal mechanistic study |
| Zimmermann et al., 2019 | In vitro and animal pharmacokinetic/mechanistic study |
| Vétizou M, 2015 | Narrative/mechanistic review |

This descriptive classification characterizes the intellectual base represented by the ten most co-cited references. It is not an OCEBM therapeutic evidence grade and is not representative of the entire contemporary evidence base.
